# Polysaccharide fraction from *Triplostegia glandulifera* Wall and its renoprotective effect in streptozotocin-induced diabetic mice by attenuating oxidative stress

**DOI:** 10.1007/s13659-024-00467-7

**Published:** 2024-08-19

**Authors:** Hai-Hui Guo, Lei Wu, Dan Mi, Xing-Yu Zhang, Fu-Mei He, Ting Lei, Fu-Sheng Wang

**Affiliations:** 1 College of Pharmacy, Dali University, Xueren Road 2, Xiaguan, Dali, 671000 Yunnan Province People’s Republic of China; 2 Yunnan Key Laboratory of Screening and Research On Anti-Pathogenic Plant Resources From Western Yunnan, Dali, 671000 People’s Republic of China

**Keywords:** *Triplostegia glandulifera*, Diabetic nephropathy, Oxidative stress, Streptozotocin, Human renal mesangial cells

## Abstract

*Triplostegia glandulifera* Wall (*T. glandulifera*) is an ethnomedicine commonly used by ethnic minorities in Yunnan, China, to treat kidney disease. However, there are few reports on the renoprotective effects of this substance, and the active ingredients remain unclear. In this study, we extracted the polysaccharide fractions TGB and TGC using the water extraction-alcohol precipitation method and determined their molecular weight (Mw) and monosaccharide composition. The study investigated the protective effects of TGB and TGC fractions against diabetic nephropathy (DN) using an in vitro high glucose-induced HRMCs model and an in vivo STZ-induced diabetic mouse model. HPLC analysis revealed that TGB contained D-galacturonic acid, D-glucose, D-galactose, and D-arabinose, and had a lower Mw than TGC. In vitro, TGB showed concentration-dependent antioxidant activity and effectively reduced abnormal proliferation and while attenuating oxidative stress in HRMCs. In mice with diabetes, TGB corrected the dysregulation of glucose-lipid metabolism and alleviated oxidative stress in the kidneys. Additionally, it improved renal function and reduced renal tissue damage. The study suggests that the low Mw polysaccharides (TGB) have better activity against DN through the antioxidative stress mechanism.

**Graphical Abstract:**

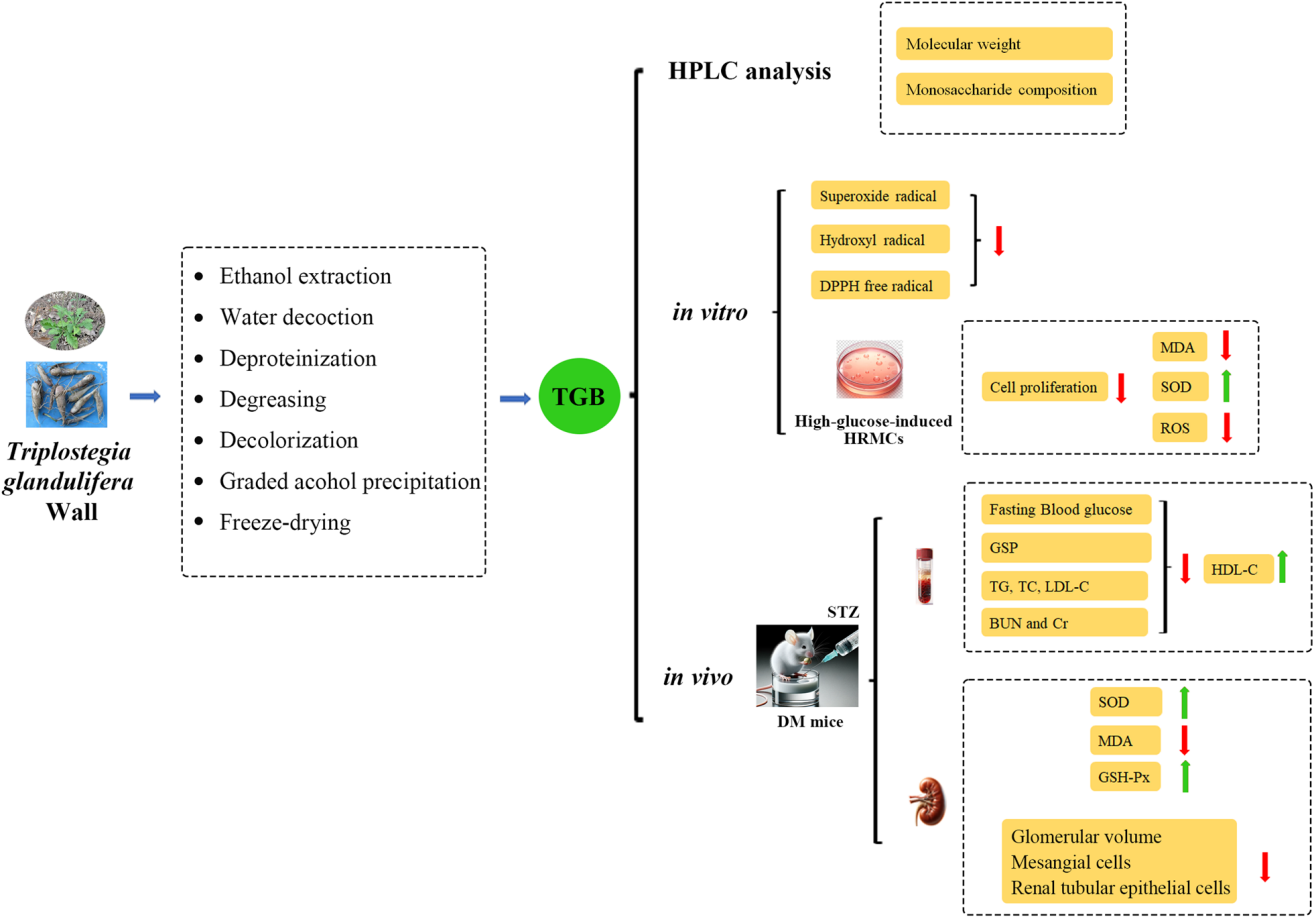

## Introduction

Diabetes Mellitus (DM) is a prevalent metabolic disorder, with its incidence continuously rising worldwide, posing a significant public health concern. The primary characteristic of diabetes is persistent hyperglycaemia, which is a key factor in the development of diabetic microvascular complications, including nephropathy [1]. Diabetic Nephropathy (DN) is a severe microvascular complication that can develop in individuals with type 1 diabetes (15%–40%) and type 2 diabetes (5%–20%) [2]. In the early stages of diabetes, there is often a phenomenon of increased glomerular filtration, which is considered a crucial factor in the progression of DN. Increasing evidence suggests that functional abnormalities of glomerular mesangial cells (MCs) play a pivotal role in the hyperfiltration observed in diabetes [3]. MCs serve as important targets of diabetic metabolic abnormalities, closely associated with structural and functional abnormalities in DN. Under hyperglycaemic conditions, MCs undergo abnormal proliferation and extracellular matrix accumulation as the disease progresses, leading to a series of structural changes in the kidneys. These changes include thickening of the glomerular basement membrane, enlargement of glomerular volume, and oedema in renal tubular epithelial cells. Such structural alterations eventually lead to proteinuria and a gradual decline in renal function [4], potentially culminating in end-stage renal disease [5]. Currently, the pharmacological treatment of DN primarily includes hypoglycaemic agents, antihypertensive drugs, inhibitors targeting the renin–angiotensin–aldosterone system, and sodium-glucose cotransporter-2 inhibitors [6]. However, these medications cannot significantly halt the progression of the disease and are associated with side effects. Therefore, it is of great value and urgency to develop new, safe and effective drugs for the treatment of DN.

Oxidative stress plays a crucial role in the pathogenesis of DN. Patients with diabetes frequently suffer from oxidative stress, which causes cellular and tissue damage and promotes disease progression. Studies indicate that consuming antioxidants can reduce oxidative stress, thereby alleviating symptoms and preventing diabetes complications [7]. Many studies have shown that the active components of water-soluble fractions from medicinal plants have antioxidative and renoprotective effects, which play a role in the treatment and prevention of DN. Among them, some of the active components of traditional herbal remedies, have been shown to attenuate DN in STZ-induced diabetic rats by anti-oxidative stress [8].

*Triplostegia glandulifera* Wall (*T. glandulifera*) is a type of medicinal plant that is commonly used by the Yi minority in southwest China [9, 10]. Its earliest record dates back to *Yunnan ZhongcaoYao Xuan* [11]. The root tubers of* T. glandulifera* are commonly used to treat kidney diseases [12]. *T. glandulifera* is taken as a poached decoction according to empirical use. Our previous study showed that the 95% ethanol extract of *T. glandulifera *was able to reduce blood glucose levels in diabetic mice by scavenging free radicals and inhibiting lipid peroxidation [13]. However, there are few reports on the renal protection of the water-soluble active components from *T. glandulifera*. Herein, the extraction and purification of water-soluble polysaccharide fractions were studied with *T. glandulifera* as the raw material. At the same time, the anti-DN activity of water-soluble polysaccharide fractions was investigated.

## Results

### Effects of TGB and TGC on antioxidant activity in vitro

**Fig. 1:**
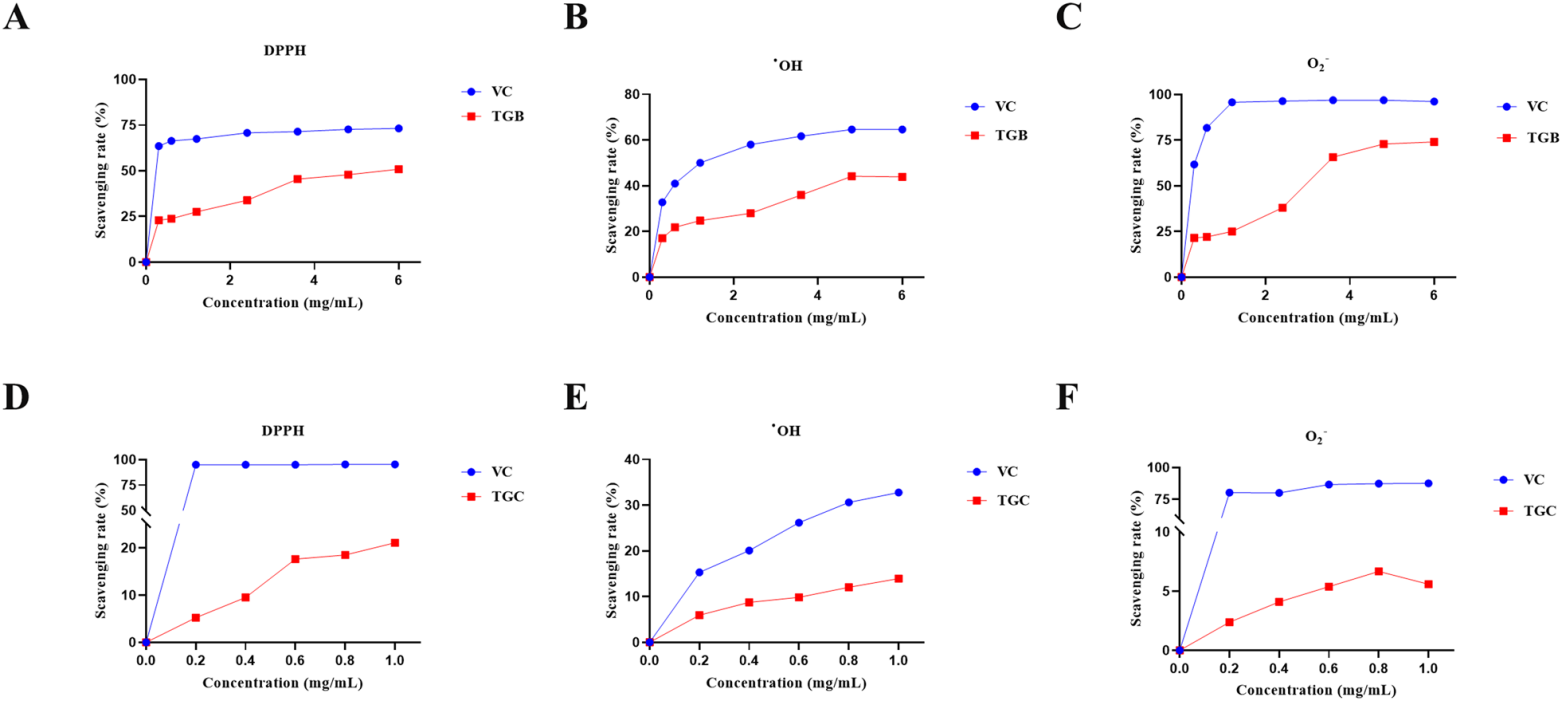
Effects of TGB and TGC on antioxidant activity in vitro. **A** DPPH free radical scavenging activity of TGB; **B** Hydroxyl radical (^•^OH) scavenging activity of TGB; **C** Superoxide anion radical (O_2_^−^) scavenging activity of TGB; **D** DPPH free radical scavenging activity of TGC; **E** Hydroxyl radical (^•^OH) scavenging activity of TGC; **F** Superoxide anion radical (O_2_^−^) scavenging activity of TGC

The evaluation of antioxidant capacity in vitro is of considerable importance and can be used as a preliminary screening method for potential antioxidant compounds in a variety of plants. As anticipated, TGB exhibited concentration-dependent scavenging rate of superoxide, hydroxyl and DPPH radicals. Similarly, TGC exhibited a mild concentration-dependent antioxidant effect (Fig. 1).

### Effects of TGB and TGC on HRMCs viability

**Fig. 2:**
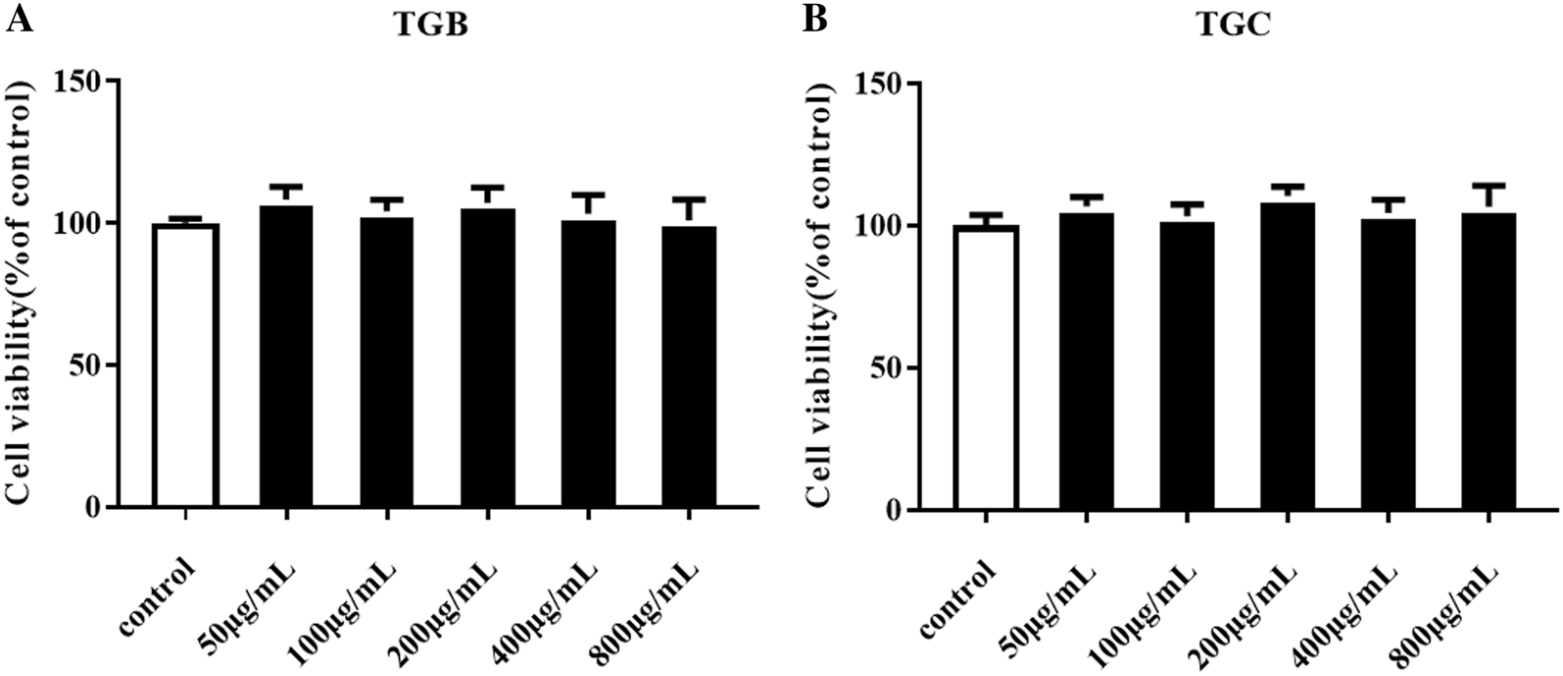
Effects of TGB and TGC on HRMC viability. **A** cell viability of TGB; **B** cell viability of TGC; Data are expressed as means ± SD, (n = 6)

HRMCs were treated with a dose range of TGB and TGC (50 to 800 μg/mL) for 24 h as described in Materials and Methods. MTT results showed that treatment with TGB and TGC in the range of 50–800 µg/mL for 48 h had no significant effect on the survival rate of HRMCs (Fig. 2).

### Effects of TGB and TGC on the proliferation of high glucose-induced HRMCs

**Fig. 3:**
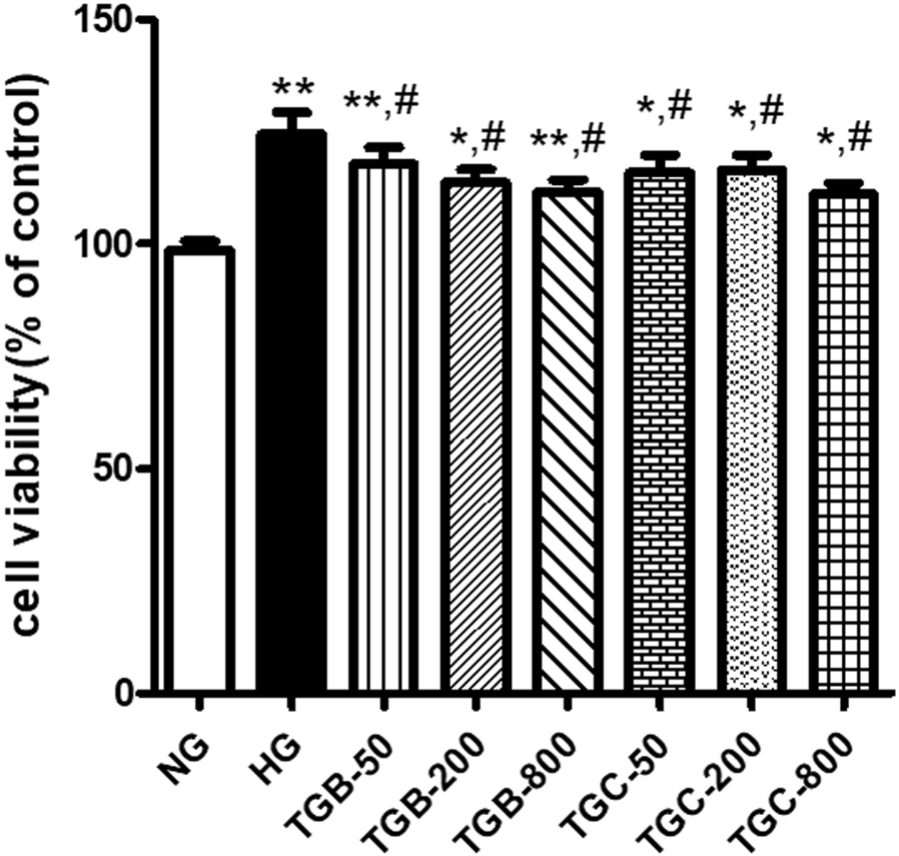
Effects of TGB and TGC on the proliferation of glucose-induced HRMCs. Cell viability of high glucose-induced HRMCs treated with TGB and TGC. TGB-50: model group was treated with TGB of 50 μg/mL; TGB-200: model group was treated with TGB of 200 μg/mL; TGB-800: model group was treated with TGB of 800 μg/mL; TGC-50: model group was treated with TGC of 50 μg/mL; TGC-200: model group was treated with TGC of 200 μg/mL; TGC-800: model group was treated with TGC of 800 μg/mL; Data are expressed as means ± SD, (n = 6). *P < 0.05, **P < 0.01 versus NG group; ^#^*P* < 0.05 versus HG group

To explore the effect of the polysaccharide fractions on DN in vitro, we assessed the impact of varying concentrations of TGB and TGC on the proliferation of HRMCs cultured in high glucose medium (Fig. 3). Compared with the NG group, abnormal proliferation of HRMCs in HG group was significantly increased (*P* < 0.05). Treatments with TGB and TGC markedly decreased HRMCs viability compared to the HG group (*P* < 0.05).

### Effects of TGB and TGC on the activities of T-SOD and the levels of MDA and ROS in high glucose-induced HRMCs

**Fig. 4:**
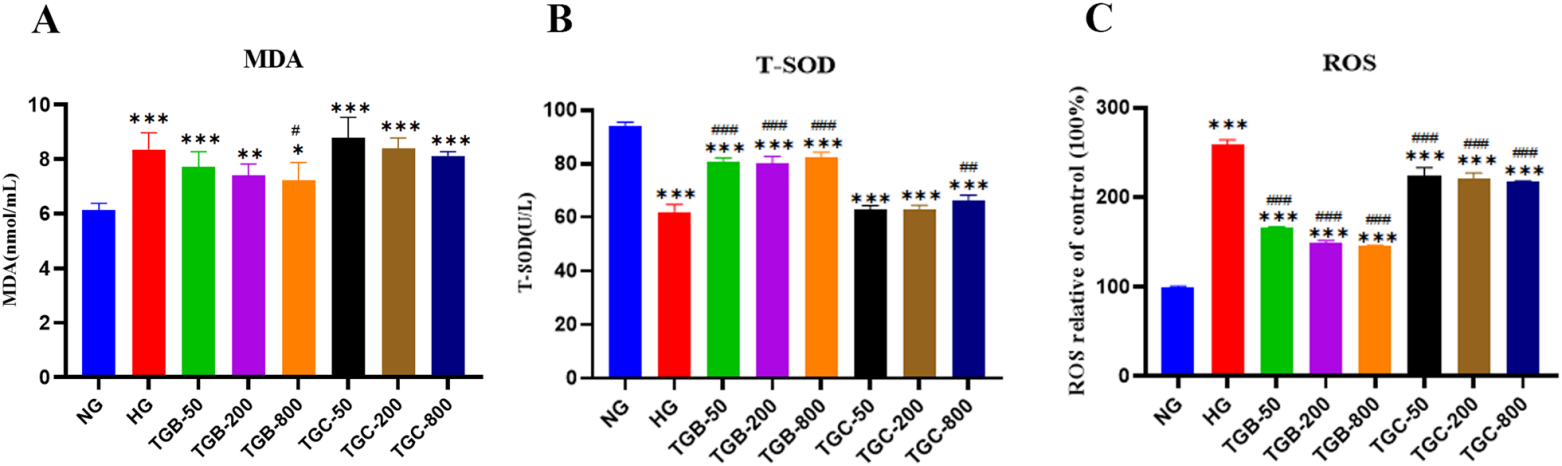
Effects of TGB and TGC on antioxidant enzyme levels in glucose-induced HRMCs. **A** MDA (malondialdehyde), **B** T-SOD (total superoxide dismutase), **C** ROS (reactive oxygen species). Data are expressed as means ± SD, (n = 6). *P < 0.05 versus NG, **P < 0.01 versus NG, ***P < 0.001 versus NG; ^#^*P* < 0.05 versus HG, ^##^*P* < 0.01 versus HG, ^###^*P* < 0.001 versus HG

To evaluate the antioxidative response in HRMCs, we quantified the levels of MDA, T-SOD and ROS as shown in Fig. 4. Compared to the NG group, the HG group displayed significantly elevated levels of MDA (*P* < 0.001) and ROS (*P* < 0.001), along with a marked reduction in T-SOD levels (*P* < 0.001), indicating increased oxidative stress in HRMCs. Notably, treatments with TGB significantly increased T-SOD levels (*P* < 0.001), with TGB additionally leading to a significant reduction in MDA (*P* < 0.05) in a concentration-dependent manner. Moreover, treatments with TGB and TGC substantially decreased ROS levels (*P* < 0.001) when compared to the HG group. The results demonstrate that the TGB can significantly alleviate oxidative stress-induced damage.

### Effect of TGB and TGC on body weight, FBG and GSP in diabetic mice

**Fig. 5:**
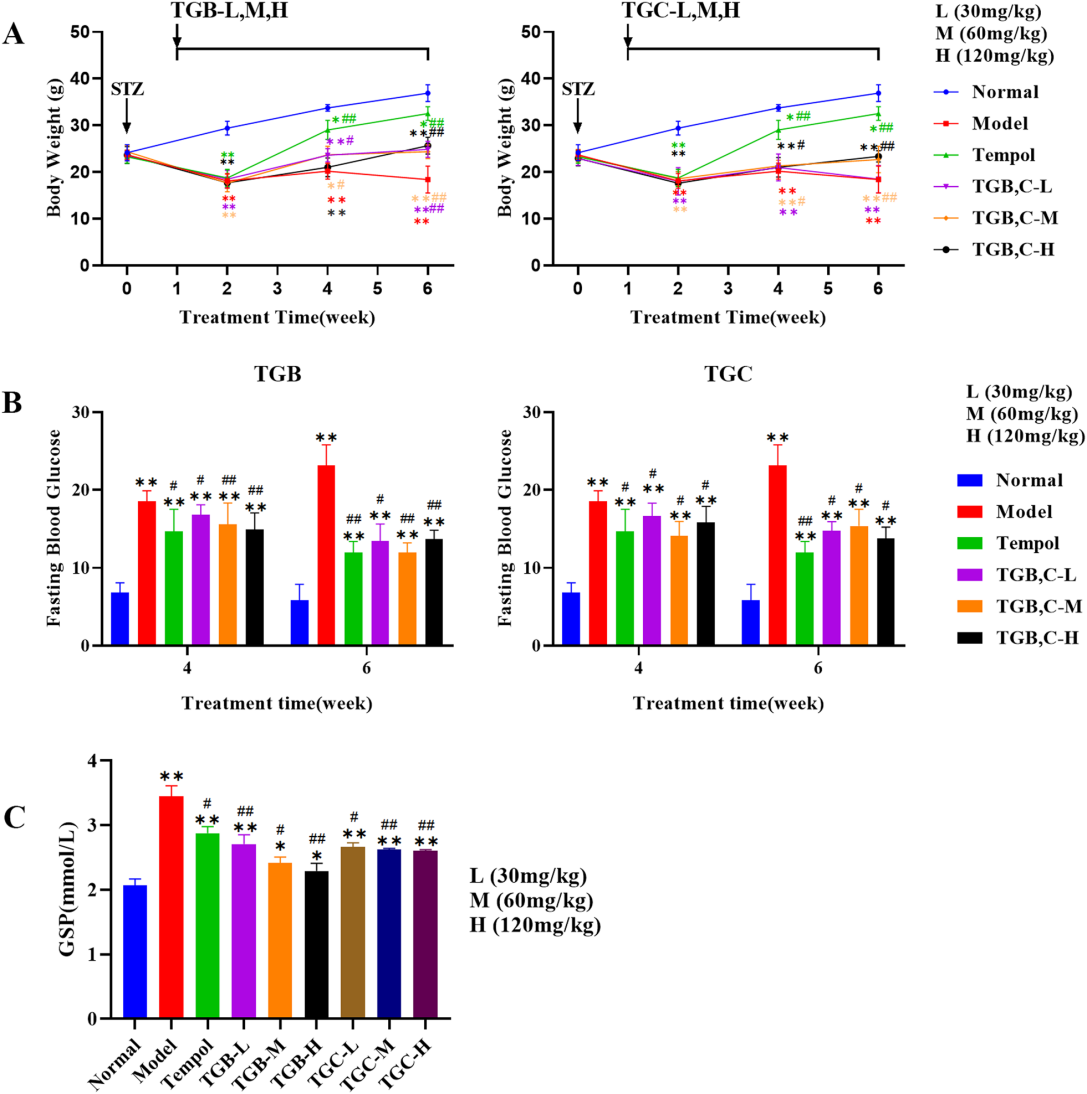
Measurement of body weight, FBG and GSP in diabetic mice induced by STZ and treatment with TGB and TGC. **A** Body weights of mice in groups L (30 mg/kg), M (60 mg/kg) and H (120 mg/kg) were measured throughout the experiment; **B** TGB and TGC intervention reduced the FBG levels in diabetic mice in the 4th and 6th weeks; **C** TGB and TGC intervention reduced the GSP levels in diabetic mice in the 6th week. Data are expressed as means ± SD, (n = 10). *P < 0.05, **P < 0.01 versus Normal; ^#^*P* < 0.05, ^##^*P* < 0.01 versus Model

Figure 5A shows the weekly recordings of mouse body weights throughout the study. Tempol, a stable and cell membrane-permeable SOD mimetic, was chosen as a positive control drug for the experiment. It has been successfully used to mitigate DN of diabetic mice [14]. Following STZ induction, a significant reduction in the body weight of diabetic mice was observed, particularly pronounced during the initial week post-model establishment, compared to the control group (*P* < 0.01). Interventions with TGB and TGC ameliorated this weight loss to some extent. Crucially, in the sixth week, mice treated with TGB-H exhibited a significantly higher body weight relative to other groups. This underscores the efficacy of TGB in counteracting the body weight loss associated with DM.

Compared to the normal group, STZ administration led to an appreciable elevation in fasting blood glucose (FBG) levels in diabetic mice (*P* < 0.01), indicative of persistent diabetes, even at the experiment’s conclusion (Fig. 5B). Initial four-week interventions of TGB and TGC yielded a minor reduction in FBG levels (*P* < 0.05 or 0.01), followed by a substantial decline by the sixth week (*P* < 0.05 or 0.01) compared to the model group. Post-six weeks, treatment with TGB-H (120 mg/kg) and TGC-H (120 mg/kg), resulted in further reductions of 36.22% and 31.21% respectively (*P* < 0.01 for TGB and *P* < 0.05 for TGC), attesting to the superior hypoglycaemic effect of TGB over TGC.

Contrasted with the normal group, a significant escalation in GSP levels was observed in the model group of mice (Fig. 5C) (*P* < 0.01). The administration of TGB and TGC resulted in a statistically significant reduction in GSP levels when compared to the model group (*P* < 0.05 or 0.01). It is worthy of note that the TGB group displayed a more pronounced reduction in effect than the Tempol group.

### Effect of TGB and TGC on serum lipids in diabetic mice

**Fig. 6:**
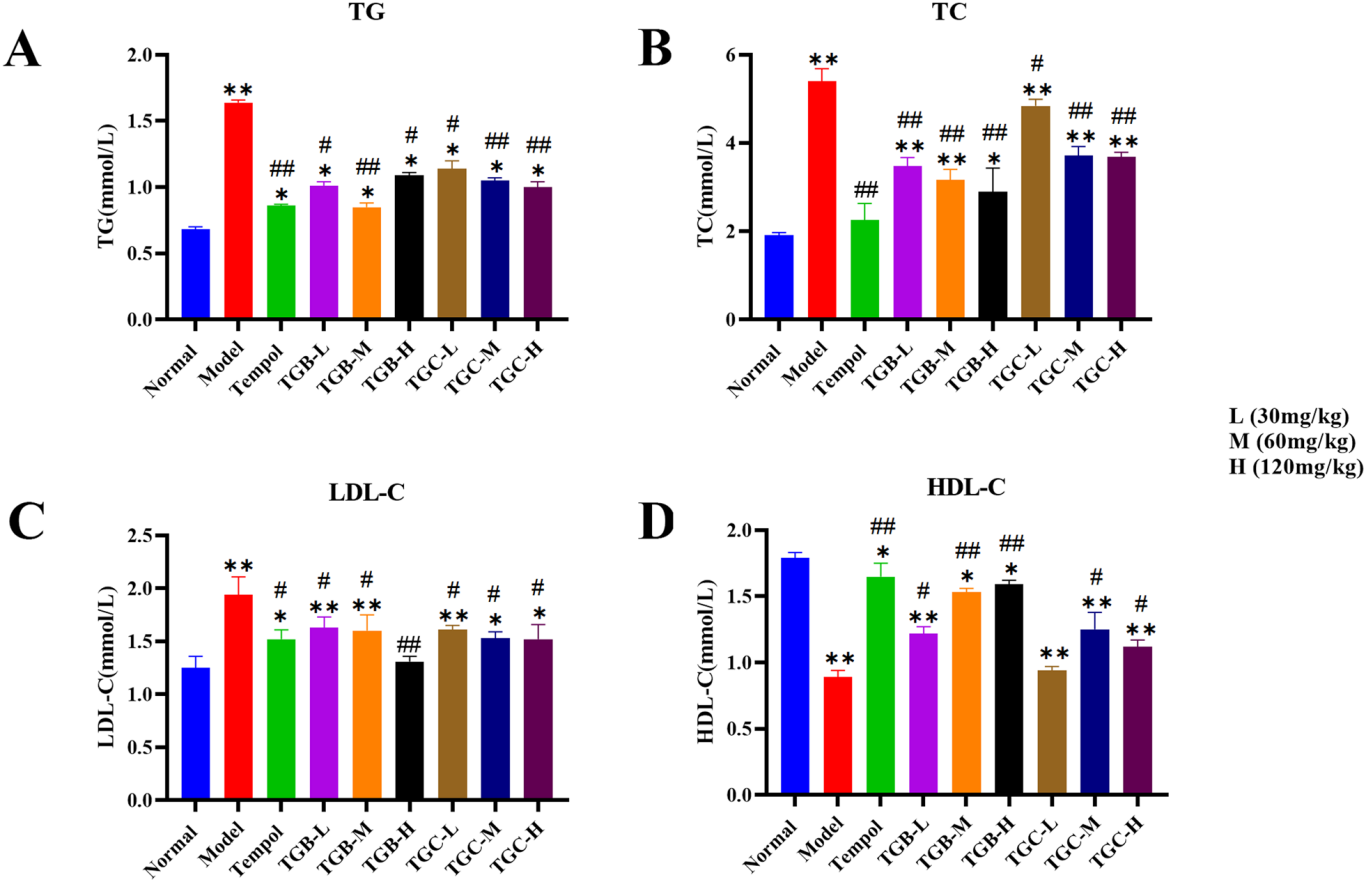
Effects of TGB and TGC on serum lipid in diabetic mice. **A** TG (triglyceride), **B** TC (total cholesterol), **C** LDL-C (low density lipoprotein), **D** HDL-C (high density lipoprotein). Data are expressed as means ± SD, (n = 10). *P < 0.05, **P < 0.01 versus Normal; ^#^*P* < 0.05, ^##^*P* < 0.01 versus Model

Dysregulated lipid metabolism is a hallmark of DM. The study concluded that serum TG, TC and LDL-C were significantly higher in diabetic mice than in the normal group, while HDL-C was significantly lower (Fig. 6A–D), highlighting the significant dyslipidemia in these mice. Intriguingly, 6 weeks of treatment with TGB and TGC considerably mitigated these lipid abnormalities, reducing TG, TC, LDL-C, and enhancing HDL-C levels. At 6 weeks, TGB administration significantly reversed these changes by 33.54%, 46.48%, 32.47% and 78.65%, respectively. Meanwhile, TGC administration resulted in significant reversals of changes by 39.02%, 31.85%, 21.65% and 25.84%. Notably, TGB-H demonstrated a superior hypolipidaemic effect compared to TGC. All observed differences were statistically significant against the model group (*P* < 0.05 or 0.01), as shown in Fig. 6.

### Effect of TGB and TGC on the renal functions of diabetic mice

**Fig. 7:**
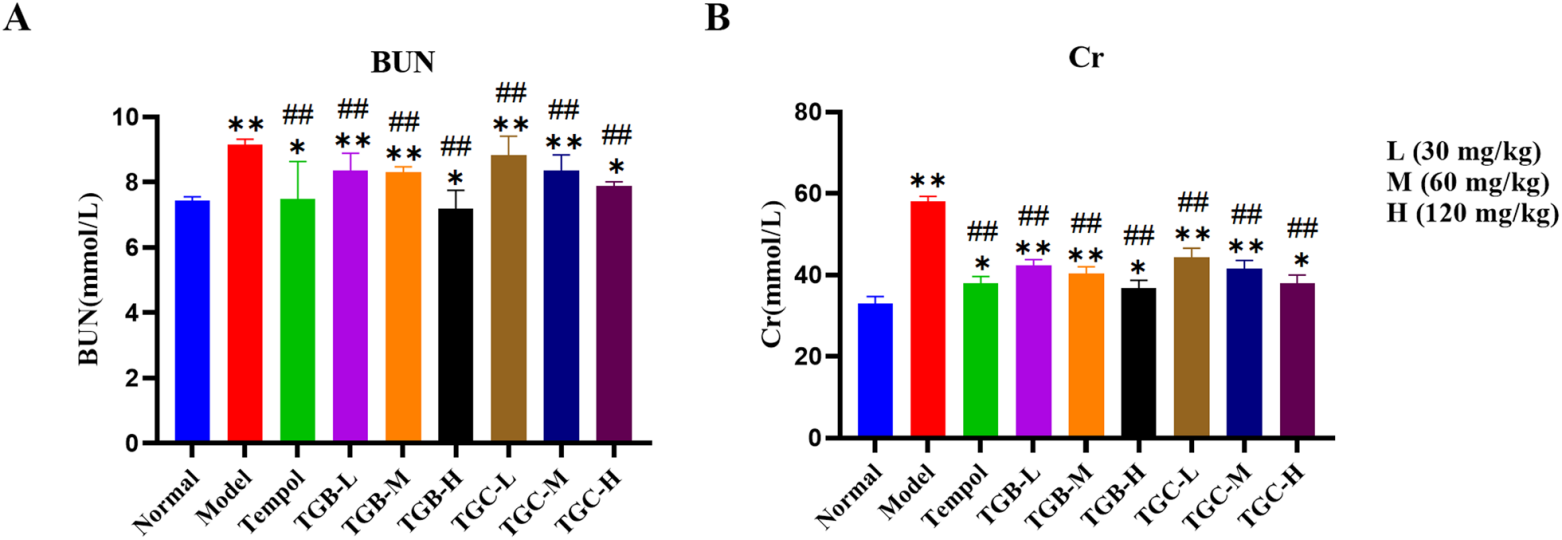
Effects of TGB and TGC on the renal functions of diabetic mice. **A** BUN (blood urea nitrogen), **B** Cr (creatinine). Data are expressed as means ± SD, (n = 10). *P < 0.05, **P < 0.01 versus Normal; ^##^*P* < 0.01 versus Model

The experimental results showed that compared with the normal group, renal function in the model group of mice was significantly changed, and STZ-induced hyperglycaemia led to a significant increase in BUN and Cr levels (Fig. 7) (*P* < 0.01). However, treatment with TGB and TGC resulted in a remarkable reduction in BUN and Cr levels in comparison to the model group, indicating a protective effect of *T. glandulifera* on renal function in DN mice (*P* < 0.05 or 0.01). At last, the BUN levels in the TGB-H group returned to normal, indicating that TGB group had the best renal function recovery.

### Effect of TGB and TGC on renal structure in diabetic mice

**Fig. 8:**
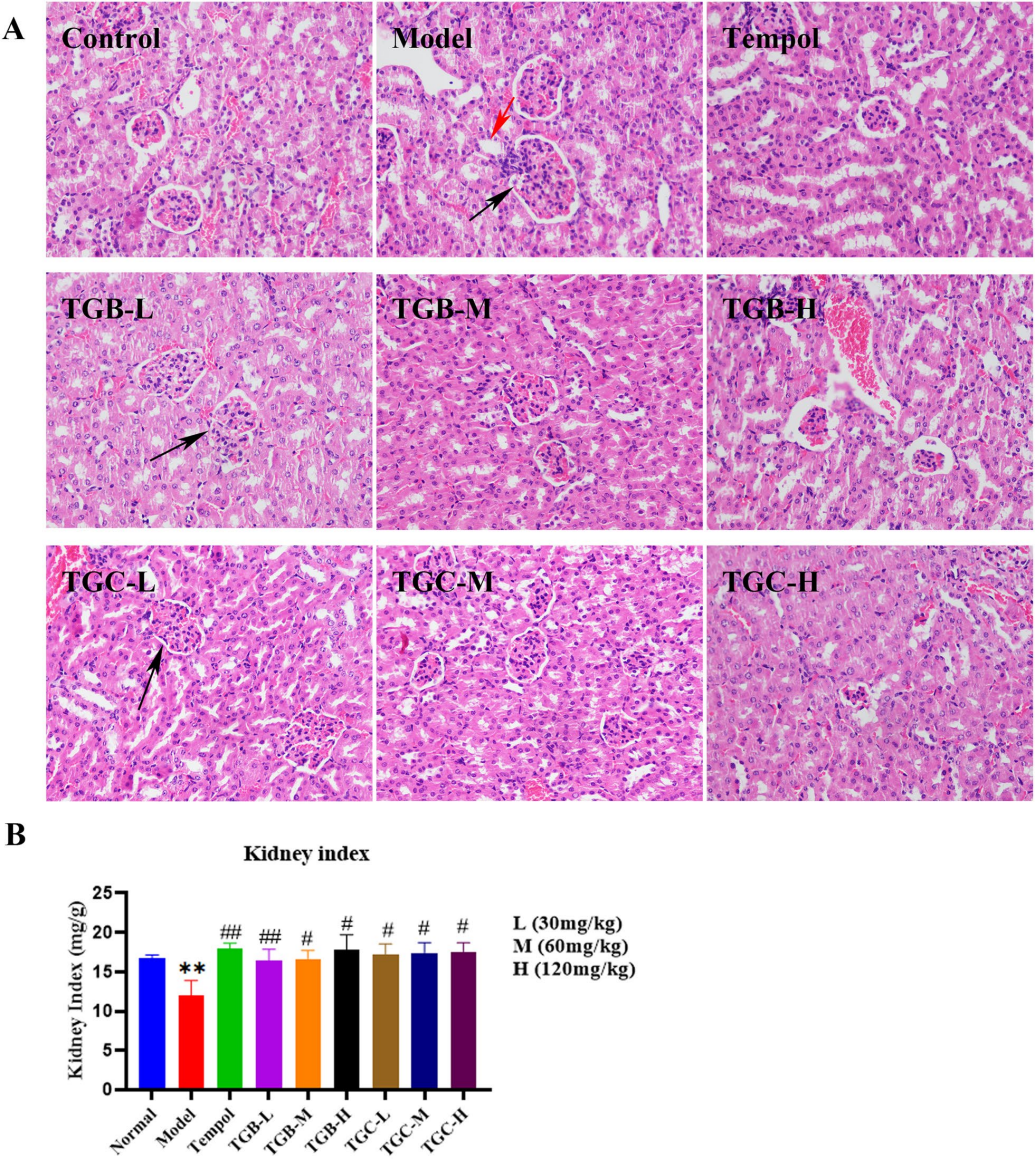
Effects of TGB and TGC on renal structure of diabetic mice. **A** Hematoxylin and eosin (H&E) sections of the kidney tissues after 6 weeks’ administration from normal mice, model mice, Tempol-treated DN mice and *T. glandulifera*-treated DN mice (L = 30, M = 60, H = 120 mg/kg/d). The black arrows in the figure denote the enlargement of the glomerular volume coupled with segmental proliferation of the MCs, and the red arrows indicates renal tubular epithelial cells oedema (magnification × 400). **B** Kidney/body weight (kidney index, percentage) was measured in the 6th weeks after administration of *T. glandulifera*. Data are expressed as means ± SD, (n = 10). **P < 0.01 versus Normal; ^#^*P* < 0.05, ^##^*P* < 0.01 versus Model

H&E staining was used for histological examination (Fig. 8A). The kidney of mice in the model group exhibited significant structural changes compared to the control group. The glomerular volume of the model group was significantly increased. Additionally, there was segmental proliferation of MCs and oedema in renal tubular epithelial cells. Notably, Tempol effectively ameliorated these pathological alterations. All other doses of TGB and TGC were effective in reducing glomerular volume and inhibiting segmental proliferation of MCs, as well as alleviating oedema in renal tubular epithelial cells.

Furthermore, the kidney index of mice in the model group was significantly lower than that of the normal group (*P* < 0.05, Fig. 8B). Fortunately, the renal indices of diabetic mice in TGB and TGC groups after treatment were not statistically different from those in the normal group (*P* > 0.05), and the TGB-H group is the closest to Tempol.

### Effect of TGB and TGC on antioxidant indexes in diabetic mice

**Fig. 9:**
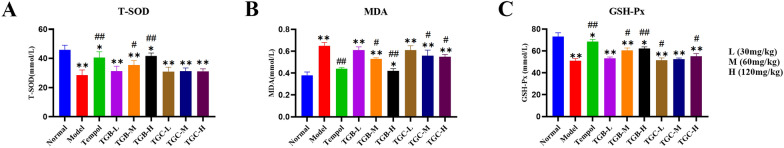
Effects of TGB and TGC on antioxidant enzymes levels in kidney of diabetic mice. **A** T-SOD (total superoxide dismutase), **B **MDA (malondialdehyde), **C** GSH-Px (glutathione peroxidases). Data are expressed as means ± SD, (n = 10). *P < 0.05, **P < 0.01 versus Normal; ^#^*P* < 0.05, ^##^*P* < 0.01 versus Model

In addition, the levels of antioxidant indicators, including T-SOD, MDA and GSH-Px in kidney, were notably changed in DM group as compared with normal group (*P* < 0.01, Fig. 9). In kidney, compared with model group, TGB-H (120 mg/kg) treatment increased the T-SOD and GSH-Px levels by 46.39% (*P* < 0.01) and 22.15% (*P* < 0.01) reduced MDA by 35.38% (*P* < 0.01), TGC-H (120 mg/kg) treatment increased the GSH-Px levels by 8.31% (*P* < 0.05) and reduced MDA by 15.38% (*P* < 0.05). Unfortunately, T-SOD levels increased by 9.54% (*P* > 0.05), which did not reach statistical significance. Furthermore, the therapeutic effects of TGB-H group on MDA and T-SOD in mouse renal tissue were superior to Tempol group. Overall, the antioxidant activity of TGB was observed to be more pronounced than that of TGC, and the TGB fraction shows a significant dose-dependent effect.

### HPLC analysis of TGB and TGC

**Fig. 10:**
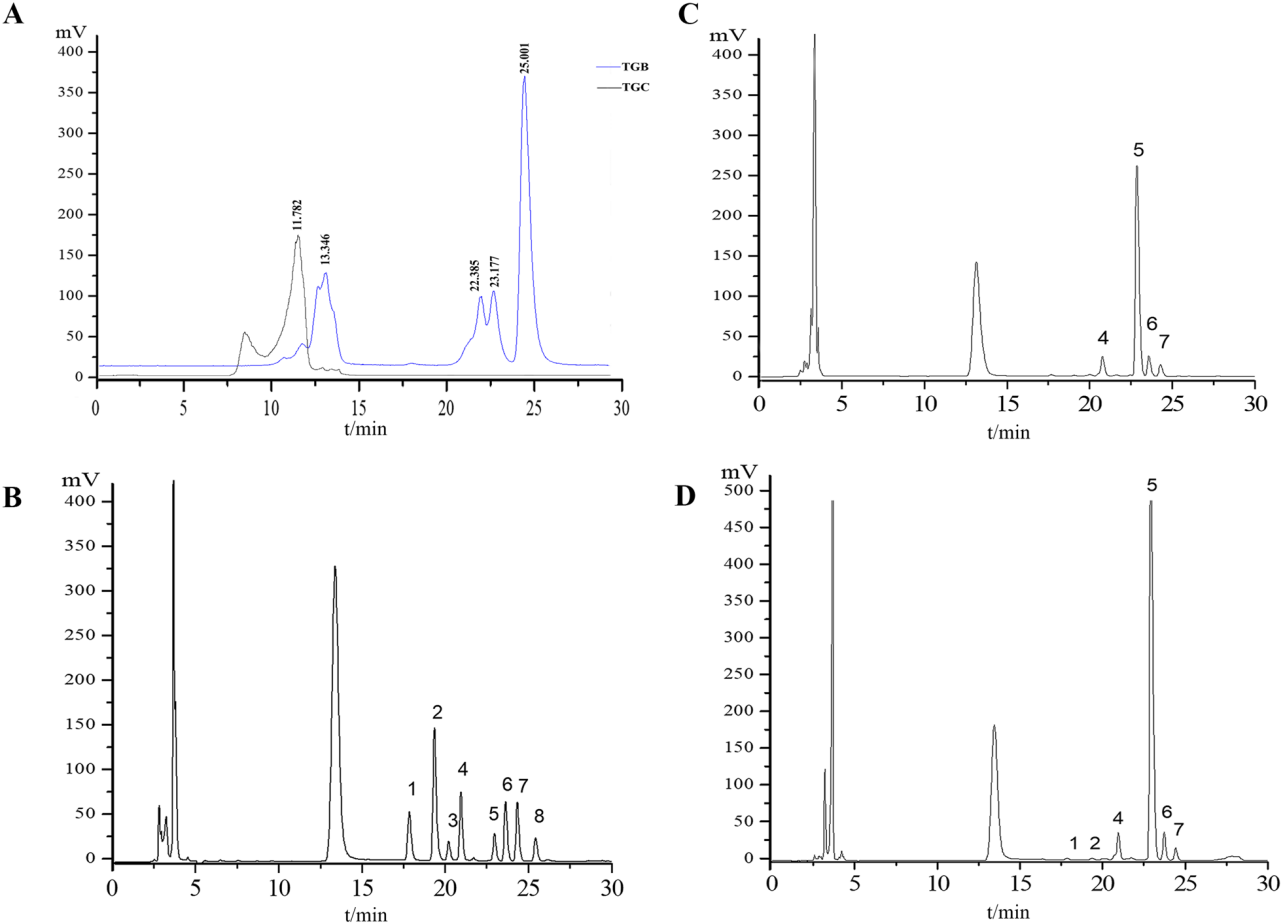
HPLC analysis of TGB and TGC. **A** Mw distribution of TGB and TGC. **B** Chromatogram of 8 standard reference substances. **C** Monosaccharide composition of TGB. **D** Monosaccharide composition of TGC. 1. D-Mannose, 2. D-Glucosamine, 3. D-Glucuronic acid, 4. D-Galacturonic acid, 5. D-Glucose, 6. D-Galactose, 7. D-Arabinose, 8. L-Fucose

Gel permeation chromatography was used to determine the Mw of polysaccharides in TGB and TGC. Using a series of Mw standards, a standard calibration curve was plotted with the logarithm of the Mw (log Mw) as the vertical axis against the RT as the horizontal axis. This resulted in a linear regression equation of y = −0.3491x + 9.9531, with a coefficient of determination (R^2^) of 0.994. On the basis of the RT observed in Fig. 10A, the main Mw of polysaccharides in TGC was 690 kDa. The Mw of polysaccharides in TGB was found to be less than 200 kDa. The monosaccharide composition of the polysaccharides was determined by comparing the spectra of the sample solution with those of the mixed monosaccharide standard solution (Fig. 10B–D). The molar ratios of monosaccharide composition (Table 1) were calculated by referencing the standard curve (Table 2). TGB has a higher proportion of galactose than TGC.

**Table 1:** The monosaccharide composition and molar ratio of TGB and TGC

Sample solution	Monosaccharide composition	Molar ratio
TGB	GalA Glc Gal Ara	0.49:5.65:0.50:0.35
TGC	Man GlcN GalA Glc Gal Ara	0.08:0.06:1.05:15.71:0.92:0.49

**Table 2:** Standard curve for monosaccharides

Monosaccharides	Regression equatio	Lineraity range(mg/mL)	Correlation coefficient R^2^
D-Mannose	y = 406.3x + 108.65	0.50–8.0	0.9995
D-Glucosamine	y = 588.62x-49.41	0.52–8.5	0.9995
D-Glucuronic acid	y = 145.22x + 45.12	0.47–7.5	0.9998
D-Galacturonic acid	y = 321.59x + 113.87	0.44–6.9	0.9999
D-Glucose	y = 191.55x + 250.95	0.44–7.0	0.9996
D-Galactose	y = 482.59x + 96.92	0.38–6.0	0.9996
D-Arabinose	y = 298.8x + 210.95	0.50–7.8	0.9997
L-Fucose	y = 126.25x + 318.95	0.56–9.0	0.9998

## Discussion

In this study, we have investigated the antioxidant activity of *T. glandulifera* water-soluble polysaccharides through in vitro and in vivo experiments. The in vitro research includes DPPH, superoxide anion, and hydroxyl radical scavenging assays, as well as HRMCs experiments. Additionally, in vivo experiments were conducted on STZ-induced diabetic mice. Phytochemical studies have concluded that TGB (the low Mw polysaccharide fraction of *T. glandulifera*) exhibits nephroprotective effects on DN by alleviating oxidative stress in a concentration-dependent manner.

*T. glandulifera* has a long history of use in folk medicine. The water decoction of this plant is effective in the prevention and treatment of DN [12]. Although *T. glandulifera* is used as an ethnomedicine by the Yi ethnic group in the southwest of China, few studies have been conducted on its activity by other research labs. Our lab's research has shown that the alcoholic extract of *T. glandulifera* has a hypoglycaemic effect on different causes of hyperglycaemia [15]. Furthermore, it displays antioxidant activity both in vivo and in vitro [13]. These experiments have shown that *T. glandulifera* is abundant in water-soluble polysaccharides. Significantly, these polysaccharides manifest no apparent toxic effects. The extraction of *T. glandulifera* was conducted sequentially using 95% ethanol and hot water. The resulting crude extract consisted of 18.6% ethanol extract and 81.4% hot water extract. Polysaccharides, recognized as the principal active constituents in diverse anti-diabetic plants, have garnered considerable attention from researchers. Actually, in China, a variety of polysaccharide products have been used as alternative medicines to treat DM in clinic, including astragalus polysaccharide, konjac glucomannan, ginseng polysaccharide, pumpkin polysaccharide and so on [16]. Therefore, finding effective anti-diabetic drugs targeting natural polysaccharides is a promising research direction. Ginseng polysaccharide showed obvious hypoglycaemic effect, and its mechanism may be related to the alleviation of oxidative stress and the increase of insulin secretion [17]. *Acanthopanax senticosus* polysaccharide had potent antioxidant activities and could attenuate the lipids profile as well as blood glucose level [18, 19]. Thus, it is a promising research direction to find effective anti-diabetic drugs focused on natural polysaccharides. In our previous study, it was found that the activity of the total polysaccharide fraction in *T. glandulifera* was significantly more effective than that of the alcohol-extracted fraction at the same dose. Sun's previous research showed that low Mw polysaccharides have better antioxidant activity than high Mw polysaccharides [20]. In this study, the active components of* T. glandulifera* were divided into two main fractions based on their Mw, specifically the high Mw polysaccharides fraction (TGC) and the low Mw polysaccharides fraction (TGB). Their respective activities in vitro and in vivo were subsequently analyzed. HPLC was used to determine the Mw, ensuring the accuracy and reliability of the method.

DN is a significant complication of DM. It is characterised by structural alterations in the kidney and a progressive loss of renal function [21]. BUN and Cr are biochemical indicators used to assess renal function. Serum Cr will not rise significantly until approximately 50% of renal function is lost [22]. The present study identified model mice with a reduced glomerular filtration rate, as evidenced by elevated levels of BUN and Cr. The TGB treatment significantly decreased BUN and Cr levels, indicating protection of renal function. TGB was more effective than Tempol in improving renal function.

Hyperglycaemia is a crucial factor in the development of DN [23]. Research indicates that TGB exhibits superior efficacy in reducing GSP levels compared to the Tempol. Tempol, a SOD mimetic, was selected as a positive control drug for the experiment. It has been successfully used to mitigate DN by alleviating oxidative stress in the kidneys of diabetic mice [14]. Additionally, it has a remarkable ability to modulate lipid dysregulation, especially exceeding Tempol in the control of TG and LDL-C. These findings suggest that TGB is effective in correcting the diabetes-induced glycolipid metabolism disorders.

The development of DN is correlated with oxidative stress induced by metabolic disorders, including hyperglycaemia and hyperlipidemia [24, 25]. These factors can damage the structure and function of the kidney. TGB treatment significantly increased T-SOD and GSH-Px levels, while MDA levels significantly decreased. TGB treatment was found to be superior to Tempol in decreasing MDA. These findings suggest that TGB may provide renal protection in diabetic mice by alleviating oxidative stress. In addition, TGB effectively inhibited the abnormal proliferation of MCs induced by high glucose, demonstrating significant renoprotective effects [6]. In both in vivo and in vitro experiments, TGB showed a mitigating effect on the high glucose-induced structural changes in the kidney, such as increased glomerular volume, proliferation of MCs, and oedema of tubular epithelial cells. The renal damage observed in diabetic mice corresponds to the early stages of renal fibrosis reported by Gu [26]. Therefore, it can serve as a reliable indicator of therapeutic efficacy. In conclusion, TGB plays a protective role in DN by alleviating oxidative stress as well as inhibiting the abnormal proliferation of MCs.

In summary, this study has shown that TGB (low Mw water-soluble polysaccharide fraction) has renoprotective effect in DN. The observed effect may be related to regulating the imbalance of glucose and lipid metabolism, enhancing the antioxidant capacity of kidney, and the maintenance of renal structure and function by TGB. Further study is required to explore the specific mechanisms. It was observed that low Mw polysaccharides had stronger nephroprotective activity in line with literature results [20]. Our experimental results revealed that low Mw polysaccharides exhibit superior water solubility compared to the high Mw fractions. This characteristic contributes to their biological efficacy [27]. The activity of polysaccharides is also influenced by the linkages between monosaccharides [28]. To gain a better understanding of the relationship between the structure of polysaccharides and their renoprotective activity, future work will involve further purification and structural characterization of TGB polysaccharides. Additionally, related proteins and signalling pathways will be explored. The scientific basis for the further development and application of *T. glandulifera* water-soluble polysaccharides will be provided by these studies.

## Materials and methods

### Chemicals and reagents

Streptozotocin (STZ) was purchased from Sigma-Aldrich Company. Blood glucose meter was purchased from Sinocare Inc (Hunan, China). ELISA kits specific for glycosylated serum protein (GSP), malondialdehyde (MDA), glutathione peroxidase (GSH-Px), blood urea nitrogen (BUN), creatinine (Cr), total superoxide dismutase (T-SOD), reactive oxygen species (ROS), triglyceride (TG), total cholesterol (TC), high-density lipoprotein cholesterol (HDL-C) and low-density lipoprotein cholesterol (LDL-C) were purchased from Nanjing Jiancheng Institute of Biotechnology (Nanjing, China). All other chemicals and reagents used in the experiments were analytical grade.

### Plant material

*T. glandulifera* root tubers were collected during November in the year 2019 from Wubao Mountain, Yunlong County, Dali Bai Autonomous Prefecture, Yunnan Province, P. R. China. The plant material was identified and authenticated by Prof. Baozhong Duan of College of Pharmacy, Dali University. The voucher specimen of *T. glandulifera* (NO. 20191126) was deposited in our lab.

### Preparation of experimental samples (TGB and TGC)

The roots of *T. glandulifera* were washed to remove debris and then shade-dried. The sliced roots (2 kg) were extracted with 95% ethanol (1:3, w/v) at room temperature for 24 h, which repeated three times. And the liquid part of the ethanol was filtered out. The residue was extracted by distilled water at 100 ℃ for 4 h, the mixture was filtered, repeated three times. The filtrate was combined. The protein was removed by Sevage method for 3 times (polysaccharide solution: chloroform: n-butanol = 1:0.4:0.1) [29]. After removing the proteins, the polysaccharide solution underwent chromatographic elution using a macroporous resin AB-8 column. The eluent used was distilled water, and the resulting eluates were collected. The aqueous solution was concentrated and mixed with anhydrous ethanol to a final concentration of 65% ethanol, then left at 4 ℃ for 12 h. The TGC was obtained by lyophilizing the precipitate. Polysaccharides in TGB were precipitated with 90% (v/v) ethanol by adding anhydrous ethanol into the supernatant solution.

### Cell culture

Human renal mesangial cells (HRMCs) were previously bought from Jennio Biotech Co., Ltd (Guangzhou, China). HRMCs were cultured in RPMI 1640 medium (Beijing Solarbio Science & Technology Co., Ltd., Beijing, China) supplemented with 10% fetal bovine serum (FBS, Gibco, CA, USA) and 1% mixed antibiotic (penicillin–streptomycin, Beijing Solarbio Science & Technology Co., Ltd., Beijing, China) at 37 ℃ with 5% CO_2_. The growth medium was replaced every 24 h.

### TGB and TGC pretreatment and determination of HRMCs viability

The TGB and TGC were dissolved in RPMI 1640 medium containing 5.5 mM glucose. The solutions were then diluted with the same medium to obtain concentrations ranging from 50 to 800 μg/mL (50, 100, 200, 400 and 800 μg/mL). HRMCs in the logarithmic growth phase were dispersed with 10% FBS containing RPMI 1640 medium after a digestion with 0.25% trypsin, and then seeded at a density of 2 × 10^4^ cells/well in a 96-well plate. The plate was incubated at 37 ℃ for 24 h in a humidified atmosphere containing 5% CO_2_. Extracts were then added to the corresponding wells and incubated for a further 48 h.

After incubation, the media in the wells were replaced with MTT reagent (Beijing Solarbio Science & Technology Co., Ltd., Beijing, China), and incubated for 4 h. Subsequently, the supernatant was discarded, and 150 μL of DMSO was added to each well. The optical density at 490 nm was measured using the Bio-Tek synergy HT microplate reader (Gene Company Limited, Wuhan, China). Cell viability was expressed by MTT optical density.

### High glucose-induced HRMCs proliferation model

After digestion and counting, HRMCs in the logarithmic growth phase were seeded at a density of 2 × 10^4^ cells/well in a 96-well plate in RPMI 1640 (with 5.5 mM glucose) supplemented with 10% FBS and 1% double antibiotic and incubated at 37 ℃ for 24 h in a humidified atmosphere containing 5% CO_2_.

The normal glucose (NG) group had a concentration of 5.5 mM, while the high glucose (HG) group had 30.0 mM. The concentration of HG was determined by pre-test. The TGB and TGC groups were treated with drug interventions at the concentrations of 50, 200 and 800 μg/mL. The culture media for all groups, except for NG, maintained a glucose concentration of 30.0 mM. The extracts were added to designated wells at concentrations of 50, 200 and 800 μg/mL. The volume of culture medium in each well was subsequently supplemented with RPMI 1640 containing the specified glucose concentrations, bringing it to a total of 100 μL. The cells were then incubated for an additional 48 h. MTT assay was conducted to detect cell viability following above instructions.

### Animals

Healthy male SPF BALB/c mice (weight 20 ± 2 g) were procured from Hunan Sja Laboratory Animal CO., Ltd (Changsha, China). Animal license No: SCXY (Xiang) 2019–0004. All animals were housed in individual polypropylene cages (10 mice/cage), reared under SPF condition (22–25 ℃, humidity of 50 ± 10%, 12 h light/12 h dark cycle), and ate and drank freely. The animals were acclimatized to the animal house for one week before experiments were performed. All the experimental protocols were strictly implemented in accordance with the Animal Care and Use Committee, and best efforts were made to minimize the pain of experimental animals.

### Diabetes induction and treatment

The model of DM mice induced by STZ was established in accordance with methodologies outlined in prior research, albeit with slight modifications [3]. The dosage for the administration of the positive control drug (Tempol, a SOD mimetic) was determined based on these established protocols [3, 14]. After a week of acclimatization, the animals were randomly divided into normal control group (n = 10), DM model group (DM, n = 80). Each mouse in the control group received 0.1 M citrate buffer (solvent), while the mouse in the model group was intraperitoneally injected with 120 mg/kg STZ prepared in 0.1 M citrate buffer as a single dose. Three days after administration, the blood of mouse was collected from caudal vein to measure FBG. Mouse with FBG level ≥ 11.1 mmol/L was considered diabetic and were used for the study.

Then, diabetic mice (FBG level ≥ 11.1 mmol/L) were selected and randomly divided into 8 groups, including the model group, Tempol group (90 mg/kg), TGB (30, 60, 120 mg/kg) groups and TGC (30, 60, 120 mg/kg) groups, with each group comprising 10 animals. Administration doses were determined by pre-tests, and 30, 60, and 120 mg/kg doses were finally applied. Next, the animals underwent continuous intragastric gavage of drugs for 6 weeks, with the control group and the model group receiving an equivalent volume of distilled water.

In addition, body weight of mice was recorded once a week. Blood samples were collected from each group of animals to measure FBG.

### Preparation of serum and kidney tissues

At the end of treatment, the mice were anaesthetized. The blood was collected from mice's eyes and centrifuged at 3000 r/min for 10 min after being left undisturbed at 25 ℃ for 60 min to obtain the serum, and stored at − 80 ℃ for further analysis. The mice were sacrificed by cervical dislocation, then the kidney tissues of the mice were quickly removed, weighed, thoroughly washed with ice-cold phosphate buffer saline (PBS, pH 7.4) and organ index was determined. The left kidney was homogenized in ice-cold PBS and stored in liquid nitrogen for further assays. The right kidney was fixed in 4% polyformaldehyde and embedded in wax for histopathological examination.

### Biochemical parameters analysis

The TG, TC, HDL-C, LDL-C, GSP, MDA, T-SOD, GSH-Px, ROS, BUN and Cr levels were determined using ELISA kits. All these indicators were measured based on the manufacturer’s guidelines.

### Histology assay

Fixed kidney tissues were embedded in paraffin, and cut into 5 μm thick slices for pathological analysis. After dewaxing and dehydration, the sections were stained with hematoxylin and eosin (H&E) for morphological analysis [26]. The stained tissues were observed through an optical microscope (Nicon, Japan) and images were taken for histopathological analysis.

### High-performance liquid chromatography (HPLC) analysis

#### Molecular weight (Mw) measurements

Gel permeation chromatography, also known as size exclusion chromatography (SEC), is used to determine the Mw of polysaccharides in TGB and TGC fractions [30]. HPLC analysis was performed by using an Agilent 1260 series HPLC system (Agilent Corp., Santa Clara, CA, USA) equipped with an Evaporative Light Scattering Detector (ELSD), and an Shodex SB-804 HQ (OHpak) column (7.5 mm × 300 mm, 10 μm, Shodex, Japan). The parameters for the ELSD were as follows: an evaporation temperature of 55 ℃, a nebulization temperature of 45 ℃, and the nitrogen flow rate of 1.3 mL/min. The mobile phase employed was ultrapure water. Flow rate: 0.5 mL/min. Column temperature: 35 ℃. Injection volume: 10 μL. The analysis samples were dissolved in water by ultrasound and filtered through 0.22 μm membrane filters before entering the HPLC system for separation.

Ten types of standard dextran, ranging from 180 Da to 2000 kDa, were used to create a control solution with a concentration of 1 mg/mL each. The samples were then dissolved into a concentration of 10 mg/mL. The standard and sample solutions were filtered and injected into the chromatographic column. The retention times (RT) were plotted against the logarithms of the Mw of the standards. Using the standard curve, the Mws of TGB and TGC were calculated using it.

#### Determination of the monosaccharide fraction of TGB and TGC

Monosaccharide composition analysis of TGB and TGC was performed by acid hydrolysis and precolumn derivatisation with 1-phenyl-3-methyl-5-pyrazolone (PMP) by HPLC as previously described [31]. Briefly, TGB (2 mg) and TGC (2 mg) were hydrolysed with 4 M trifluoroacetic acid (TFA) (1 mL) at 110 ℃ for 8 h, and the excess acid was completely removed with distilled water. The hydrolysed product and monosaccharide standards were derived using 0.5 M PMP methanol solvent and 0.3 M NaOH solution at 70 ℃ for 1 h, followed by neutralisation with 0.3 M HCl and evaporation. The derived products were detected by HPLC equipped with a DAD detector at 245 nm and Zorbax Eclipse XDB-C18 (4.6 mm × 250 mm, particle size 5 μm, Agilent Technologies, CA, USA). The mobile phase consisted of acetonitrile (A) and phosphate buffer solution (B). Elution was performed at a flow rate of 1.0 mL/min at 30 ℃ with a gradient in the range of 15:85–30:70 (A:B) from 0 to 30 min. The injection volume was 20 μL. Samples were compared to eight standard sugars, including D-glucuronic acid, D-glucosamine, D-arabinose, D-glucose, D-galactose, D-mannose, D-galacturonic acid, and L-fucose, to identify their monosaccharide composition. Regression equations and correlation coefficients were derived for standard monosaccharides by plotting the injection concentration on the x-axis and the peak area on the y-axis. The relative molar ratios of the monosaccharides were calculated from standard curves.

### In-vitro antioxidant assay

DPPH Assay. A method based on that of Kao T.H. was modified [32]. Each sample solution (1 mL) was mixed with 150 μL of a methanolic DPPH solution (0.65 mM). After mixing thoroughly, the mixture was left in the dark for 30 min and the absorbance was measured at 517 nm. The positive control used the same concentration of VC solution, while distilled water was used as the blank control.

Hydroxyl radical Assay. Detection using the Fenton method [33]. The reaction mixture contained 1 mL of 6 mM ferrous sulfate solution, 1 mL of 6 mM salicylic acid solution and 1 mL of the polysaccharide solution, and the reaction was initiated by addition of 1 mL of 6 mM hydrogen peroxide. After incubation at 37 ℃ for 1 h and cooling to room temperature, the absorbance of the reaction was measured at 510 nm and the hydroxyl radical scavenging activity was calculated as described. The positive control used the same concentration of VC solution, while distilled water was used as the blank control.

Superoxide anion radical Assay. The superoxide anion scavenging activities of TGB and TGC were assessed according to the modified method of Li [33]. In brief, 1 mL of polysaccharide solution, 4.5 mL of Tris–HCl buffer (pH = 8.2, 0.05 mol/L), and 0.4 mL of 25 mM pyrogallol solution was added to initiate the reaction. After mixing thoroughly, the mixture was left at 25 ℃ for 5 min. At last, the reaction was terminated by addition of 1 mL of 8 mM HCl, and the absorbance was measured at 299 nm. The positive control used the same concentration of VC solution, while distilled water was used as the blank control.

The scavenging effect (%) was calculated as follows:

scavenging effect (%) = (A_blank_-A_sample_)/A_blank_.

### Statistical analysis

All data are from over three independent repeated experiments and processed by GraphPad Prism 8.0 (GraphPad Software Inc., San Diego, CA, USA). The results are presented as mean ± standard deviation (SD). For the statistical analyses, one-way analysis of variance (ANOVA) was used followed by Tukey’s post hoc test, *P* < 0.05 was considered significantly different.

## Data Availability

All relevant data are within the manuscript.

## Abbreviations

BUN: Blood urea nitrogen
Cr: Creatinine
DM: Diabetes mellitus
DN: Diabetic nephropathy
FBG: Fasting blood glucose
FBS: Fetal bovine serum
GSH-Px: Glutathione peroxidase
GSP: Glycosylated serum protein
HDL-C: High density lipoprotein cholesterol
HRMCs: Human renal mesangial cells
LDL-C: Low density lipoprotein cholesterol
MCs: Glomerular mesangial cells
MDA: Malondialdehyde
Mw: Molecular weight
ROS: Reactive oxygen species
RT: Retention time
SPF: Specific pathogen-free
STZ: Streptozotocin
TC: Total cholesterol
TG: Triglyceride
*T. glandulifera*: *Triplostegia glandulifera* Wall
TGB: 90% Ethanol precipitate from the aqueous extract of *T. glandulifera*
TGC: 65% Ethanol precipitate from the aqueous extract of *T. glandulifera*
T-SOD: Total superoxide dismutase
